# Contributions of the EURO 2020 football championship events to a third wave of SARS-CoV-2 in Scotland, 11 June to 7 July 2021

**DOI:** 10.2807/1560-7917.ES.2021.26.31.2100707

**Published:** 2021-08-05

**Authors:** Kimberly Marsh, Emily Griffiths, Johanna J. Young, Carrie-Anne Gibb, Jim McMenamin

**Affiliations:** 1 Public Health Scotland, Glasgow, Scotland

**Keywords:** COVID-19, SARS-CoV-2, coronavirus, surveillance, mass gatherings, sporting events, public health response

## Abstract

Public Health Scotland used Scottish national contact tracing data to estimate the European football championship (EURO 2020) contributions to a third wave of SARS-CoV-2 infections. From 11 June to 7 July 2021, 2,632 (4%) of 63,874 SARS-CoV-2 cases self-reported attending a EURO 2020 event; 90% were male, of whom 73% were 20–39-year-olds. Most cases attended unofficial gatherings and averaged more contacts than the general population. Targeted guidance on celebrating safely in closed spaces is key.

In early May 2021, Scotland entered a third wave of severe acute respiratory syndrome coronavirus 2 (SARS-CoV-2) infections. This wave was characterised by a 20-fold increase in diagnosed cases. The rapid rise, from a 7-day cumulative incidence of 21 per 100,000 population to 427 cases per 100,000 population from 4 May to 3 July 2021, occurred alongside several notable events. These included the Delta variant (Phylogenetic Assignment of Named Global Outbreak (Pango) lineage designation B.1.617.2) overtaking Alpha (B.1.1.7) as the dominant strain circulating in Scotland [1], a gradual relaxation of lockdown restrictions [2] and the 2020 European football championship (EURO 2020). We used contact tracing data routinely collected through telephone interviews, undertaken as part of the ‘Test and Protect’ system implemented by the Scottish government [3], to describe the potential contributions of EURO 2020 to a third wave of SARS-CoV-2 in Scotland.

## Scotland’s participation in and recording of the EURO 2020 events in the contact tracing system 

From 11 June to 11 July 2021, Scotland and 10 other countries across Europe hosted 51 EURO 2020 matches. The Scottish football team participated in two matches at Hampden Park in Glasgow on 14 June and on 22 June. A third match took place at London’s Wembley Stadium on 18 June 2021. Around 20,000 of the 5.5 million people resident in Scotland were reported to have travelled to London for the game, of whom 2,600 were allocated tickets into the Wembley Stadium [4].

Prior to the start of EURO 2020, we created 10 standard tags in the ‘Test and Protect’ contact tracing system to track attendance at EURO 2020 events among those testing positive for SARS-CoV-2. Interviewers were instructed to attach all relevant tags to a case's record whenever attendance was reported at a EURO 2020 event during the individual's infectious period. The infectious period in Scotland is defined as 2 days prior to and 9 days after symptom onset (or a positive test result if asymptomatic) for a total of 12 days. However, there is strong public messaging and an expectation that once diagnosed, cases will isolate and any non-household contact exposure thereafter is minimal.

## Characteristics of Scottish SARS-CoV-2-positive cases during EURO 2020

**Figure 1:**
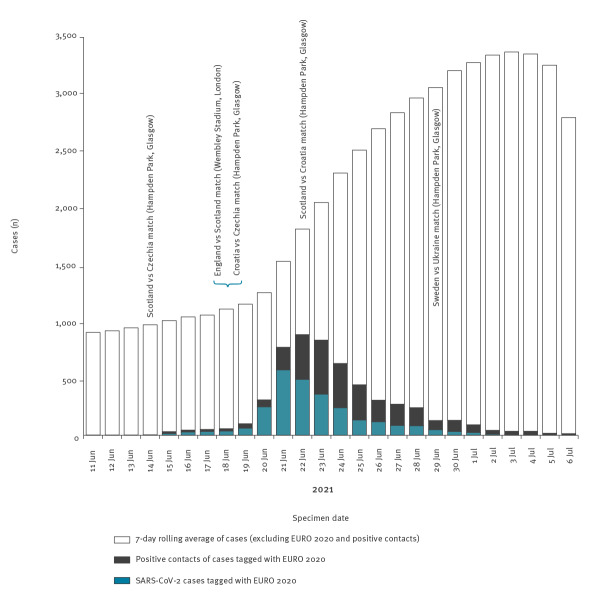
Number of SARS-CoV-2-positive cases by specimen or contact date, Scotland, 11 June–7 July 2021 (n = 63,874)

Using data abstracted from contact tracing interviews from 11 June to 7 July 2021, we identified 2,632 cases who self-reported attendance at EURO 2020-related events. The peak in cases occurred on 21 June 2021, 3 days after the England vs Scotland match at the Wembley Stadium (Figure 1) and declined gradually following Scotland’s elimination from the championship on 22 June 2021. Approximately 4% of the 63,874 new SARS-CoV-2 cases reported in Scotland from 11 June to 7 July were related to EURO 2020. Travelling to London was reported by 61% of the 2,632 cases.

SARS-CoV-2: severe acute respiratory syndrome coronavirus 2.

**Figure 2:**
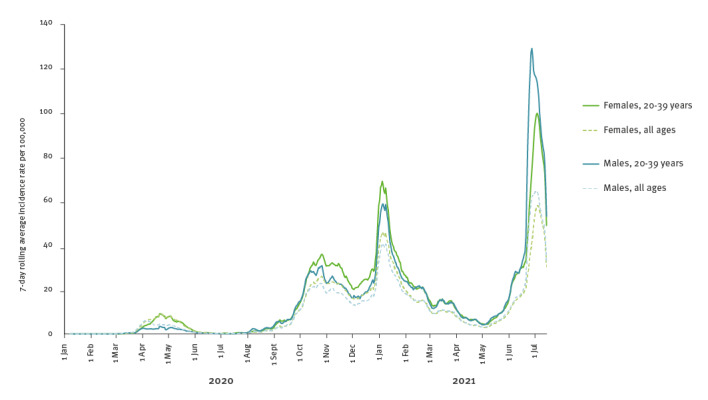
Wave dynamics of SARS-CoV-2-positive cases, 7-day rolling average incidence rate per 100,000 for all ages and ages 20–39 years, by sex, Scotland, 12 February 2020–14 July 2021(n = 323,933 cumulative SARS-CoV-2-positive cases)

Public health messages about how to safely enjoy EURO 2020 games contrasted sharply with media coverage in Scotland showing large gatherings, especially of young men, attending EURO 2020-related events. Overall, 90% of the positive cases stemming from these events were male and 73% were aged 20–39 years (Table 1). Similar age and sex patterns were observed in Scottish national data during and shortly after the EURO 2020 matches; this is different from the previous two SARS-CoV-2 waves where incidence rates among women typically exceeded those among men (Figure 2) [5].

**Table 1:** SARS-CoV-2-positive cases stemming from EURO 2020 events (n = 2,632) compared with all national infections (n = 63,874) by age and sex, Scotland, 11 June–7 July 2021

	SARS-CoV-2-positive cases related to EURO 2020								All infections from national contact tracing data							
	Male		Female		Unknown		Total		Male		Female		Unknown		Total	
Age group (years)	n	%	n	%	n	%	n	%	n	%	n	%	n	%	n	%
0–5	0	0	2	1	0	0	2	0	974	3	988	3	2	0	1,964	3
6–19	351	15	43	17	0	0	394	15	8,007	24	7,676	26	31	7	15,714	25
20–39	1,738	73	148	58	1	100	1,887	72	16,811	50	12,918	44	246	54	29,975	47
40–59	235	10	54	21	0	0	289	11	6,308	19	6,354	22	128	28	12,790	20
60–79	50	2	10	4	0	0	60	2	1,565	5	1,346	5	9	2	2,920	5
≥ 80	0	0	0	0	0	0	NA	0	212	1	228	1	1	0	441	1
Unknown	0	0	0	0	0	0	NA	0	17	0	17	0	36	8	70	0
**Total**	**2,374**	**100**	**257**	**100**	**1**	**100**	**2,632**	**100**	**33,894**	**100**	**29,527**	**100**	**453**	**100**	**63,874**	**100**

NA: not available; SARS-CoV-2: severe acute respiratory syndrome coronavirus 2.

SARS-CoV-2: severe acute respiratory syndrome coronavirus 2.

We identified 2,970 separate events or tags related to the 2,632 SARS-CoV-2 infections from Scotland who attended EURO 2020 (Table 2). Attendance at a hospitality venue (e.g. a pub to watch a live match) was reported by 997 (34%) cases. A review of free-text notes collected as part of the contact tracing process of 923 (31%) cases that were tagged in the contact tracing system as ‘EURO other settings’ revealed different travel modes to attend EURO 2020 events in London, most frequently by train, followed by private cars, flights and buses. 

**Table 2:** Case definitions and numbers of SARS-CoV-2-positive cases related to EURO 2020 tags coded by Scotland’s contact tracing teams, Scotland, 11 June–7 July 2021 (n = 2,970)

Tag in the contact tracing system for SARS-CoV-2-positive cases related to EURO 2020	Case definition	Frequency	
		n	%
EURO hospitality	Attended hospitality setting associated with EURO 2020 (e.g. pub showing a live match)	997	33.6
EURO other settings	Attended a gathering from 11 June to 11 July. This tag was used when the case/contact attended a gathering associated with EURO 2020 that did not take place in a football stadium, hospitality setting, fan zone, or house party (indoors/outdoors).	923	31.1
EURO attended England vs Scotland match Wembley Stadium, London, 18 June 2020	Attended England vs Scotland match at Wembley Stadium, London, 18 June 2021	452	15.2
EURO attended house party	Attended a private residence for an event associated with EURO 2020 (e.g. a house party to watch a match)	239	8.0
EURO attended Scotland vs Croatia match, Hampden Park, Glasgow, 22 June 2021	Attended Scotland vs Croatia match at Hampden Park, Glasgow, 22 June 2021	131	4.4
EURO fan zone Glasgow Green	Attended fan zone in Glasgow Green from 11 June to 11 July 2021	84	2.8
EURO attended match, Wembley Stadium, London	Attended any match other than the England vs Scotland match on 18 June 2021	59	2.0
EURO attended Scotland vs Czechia match, Hampden Park, 14 June 2021	Attended Scotland vs Czechia match at Hampden Park, Glasgow, 14 June 2021	48	1.6
EURO attended Croatia vs Czechia match, Hampden Park, 18 June 2021	Attended Croatia vs Czechia match at Hampden Park, Glasgow, 18 June 2021	26	0.9
EURO attended Sweden vs Ukraine match, Hampden Park, 29 June 2021	Attended Sweden vs Ukraine match at Hampden Park, Glasgow, 29 June 2021	11	0.4

SARS-CoV-2: severe acute respiratory syndrome coronavirus 2.

Attendance at the Scotland vs England match at the Wembley Stadium was the most commonly recorded official EURO 2020 event, with 452 (15%) cases who reported attending. Two hundred and sixteen cases (7%) reported attending four EURO 2020 matches held at Hampden Park in Glasgow, including the two in which the Scottish football team played.

## Numbers of close contacts and secondary attack rates 

Using contact tracing data, it was possible to estimate average number of contacts and secondary attack rates. The EURO 2020 index cases reported on average 5.6 close contacts vs 3.2 in the general population between 11 June and 7 July 2021. Secondary attack rates were significantly higher for EURO 2020 index cases (27.2%) than non-EURO 2020 cases (24.1%) (Z-score 6.99; p < 0.00001). On 22 June, an estimated 51% of all Scottish SARS-CoV-2-positive cases reported attendance at a EURO 2020 event or were close contacts of someone who had attended (Figure 1). 

Six days later, Scotland reported its highest number of daily cases ever of 3,930 [5]. Since 3 July 2021, the 7-day rolling average case numbers suggest that Scotland’s third wave is receding.

## Discussion

This analysis of the still-evolving third wave of SARS-CoV-2 in Scotland that coincided with the EURO 2020 games illustrates the potential impact of large sporting events on the trajectory of a country’s epidemic. At its peak, more than half of the cases reported in Scotland either attended a EURO 2020 event or were close contacts of someone who had attended. This is not surprising, given that the country’s return to the Union of European Football Associations EURO competition after 23 years offers an opportunity for celebration. 

Our results suggest that the steep increase in cases among men aged 20–39 years probably occurred as a result of more frequent social gatherings surrounding the EURO 2020 matches, rather than from official EURO 2020 events [6]. At official EURO matches and fan zones, efforts were made to ensure proper ventilation and encourage social distancing. In the case of the Wembley Stadium, attendees were required to show a negative lateral flow device test result within 48 h of the time the stadium gates were opened or proof of full vaccination (received at least 14 days before the match) before being allowed entry into the stadium. These measures probably decreased the risk of transmission of SARS-CoV-2 during official events. Data confirming this hypothesis are not yet available.

Higher case rates among the younger male population also likely occurred as a result of low vaccination coverage in this age group. At the time of the EURO 2020 games, around 19% of men aged 18–29 years and 30% of men aged 30–39 years were fully vaccinated [7]. Vaccination coverage was also lower among men compared with women in both age groups. Compared with previous SARS-CoV-2 waves, case rates among men exceeded those among women during the third wave, especially among younger men aged 20–39 years.

Critically, our study showed that EURO 2020 cases had a higher average number of contacts and a higher secondary attack rate than the general population. The majority of the cases reported attending unofficial EURO 2020 events linked to smaller gatherings such as house parties, visits to pubs and restaurants, as well as extended travel highlighting a need for targeted guidance on how to safely celebrate in small informal gatherings with appropriate social distancing, proper ventilation and mask wearing in closed spaces. Evidence of the potential risks in transmitting SARS-CoV-2 in vehicles is especially well documented [8], yet travel to London by private cars and public buses were common.

While this analysis suggests that the behaviour and events surrounding EURO 2020 games (as opposed to match attendance itself) uniquely contributed to Scotland’s third COVID-19 wave, causality cannot be proven. To do so, similar information about behaviours of the uninfected population related to participation at EURO 2020 events is needed. A similar picture of rising cases has emerged elsewhere in Europe where games have been held, with a 10% increase in cases in the week leading up to 27 June 2021 [9]. The EURO 2020-related transmissions have also been documented in Finland, where 947 new SARS-CoV-2-positive cases were linked to travel to the host city Moscow, Russia [10].

It is not possible to say whether EURO 2020 cases acquired or transmitted infection while attending a specific event, especially in light of rising background prevalence. The EURO 2020 games occurred alongside other events in Scotland that could have contributed to a rise in cases, including the introduction of the potentially more transmissible SARS-CoV-2 Delta variant and an easing of lockdown measures just before the matches. 

Because this analysis uses self-reported data and some people may be reluctant to admit risky behaviours, the number of EURO 2020 cases reported is likely to be an underrepresentation of the actual number. At the same time, PCR testing uptake in Scotland increased by more than 50% from May to July 2021 [11], thus increasing case detection rates during this third wave. Further work to establish linkages between EURO 2020 and non-EURO 2020 cases using genetic sequencing are underway.

## Conclusion

Our results suggest a clear link between the increase in SARS-CoV-2-positive cases among men aged 20−39 years and the EURO 2020. The behaviour surrounding attendance at EURO 2020-related events rather than match attendance itself may have uniquely contributed to Scotland’s third wave. Increased social mixing and travel to London surrounding the games is likely to have increased cases among young men, who currently have lower vaccination coverage than the older population. Public health messaging that acknowledges the unique types of risks surrounding these events and educates people about how to best manage them is critical when planning for future large sporting events. Early exit of the Scottish football team from the EURO 2020 may have contributed to the subsequent reduction in cases that are now being observed.
